# lncRNA-mRNA Co-Expression and Regulation Analysis in Lung Fibroblasts from Idiopathic Pulmonary Fibrosis

**DOI:** 10.3390/ncrna10020026

**Published:** 2024-04-17

**Authors:** Armando López-Martínez, Jovito Cesar Santos-Álvarez, Juan Manuel Velázquez-Enríquez, Alma Aurora Ramírez-Hernández, Verónica Rocío Vásquez-Garzón, Rafael Baltierrez-Hoyos

**Affiliations:** 1 Laboratorio de Fibrosis y Cáncer, Facultad de Medicina y Cirugía, Universidad Autónoma Benito Juárez de Oaxaca, Ex Hacienda de Aguilera S/N, Sur, San Felipe del Agua, Oaxaca C.P. 68020, Mexico; armandoloopez37@cecad-uabjo.mx (A.L.-M.); jovitocesarsa@cecad-uabjo.mx (J.C.S.-Á.); juanmanuelvela_enriquez@cecad-uabjo.mx (J.M.V.-E.); aramih_09@cecad-uabjo.mx (A.A.R.-H.); veronicavasgar@gmail.com (V.R.V.-G.); 2 CONACYT-Facultad de Medicina y Cirugía, Universidad Autónoma Benito Juárez de Oaxaca, Ex Hacienda de Aguilera S/N, Sur, San Felipe del Agua, Oaxaca C.P. 68020, Mexico

**Keywords:** LncRNA, mRNA, meta-analysis, lung fibroblasts, idiopathic pulmonary fibrosis

## Abstract

Idiopathic pulmonary fibrosis (IPF) is a progressive lung disease marked by abnormal accumulation of extracellular matrix (ECM) due to dysregulated expression of various RNAs in pulmonary fibroblasts. This study utilized RNA-seq data meta-analysis to explore the regulatory network of hub long non-coding RNAs (lncRNAs) and messenger RNAs (mRNAs) in IPF fibroblasts. The meta-analysis unveiled 584 differentially expressed mRNAs (DEmRNA) and 75 differentially expressed lncRNAs (DElncRNA) in lung fibroblasts from IPF. Among these, BCL6, EFNB1, EPHB2, FOXO1, FOXO3, GNAI1, IRF4, PIK3R1, and RXRA were identified as hub mRNAs, while AC008708.1, AC091806.1, AL442071.1, FAM111A-DT, and LINC01989 were designated as hub lncRNAs. Functional characterization revealed involvement in TGF-β, PI3K, FOXO, and MAPK signaling pathways. Additionally, this study identified regulatory interactions between sequences of hub mRNAs and lncRNAs. In summary, the findings suggest that AC008708.1, AC091806.1, FAM111A-DT, LINC01989, and AL442071.1 lncRNAs can regulate BCL6, EFNB1, EPHB2, FOXO1, FOXO3, GNAI1, IRF4, PIK3R1, and RXRA mRNAs in fibroblasts bearing IPF and contribute to fibrosis by modulating crucial signaling pathways such as FoxO signaling, chemical carcinogenesis, longevity regulatory pathways, non-small cell lung cancer, and AMPK signaling pathways.

## 1. Introduction

Idiopathic pulmonary fibrosis (IPF) constitutes a chronic, progressive, and irreversible disease of still unknown etiology [1]. It is predominantly observed in adults over 60, with a higher incidence in males. This disease carries a high mortality rate, with a life expectancy of 3 to 5 years after diagnosis [1,2,3]. This is because IPF is usually detected in advanced stages, as it is generally asymptomatic in its early stages, lacks distinctive biomarkers, and has limited therapeutic options [4,5] Currently, only two antifibrotic drugs—pirfenidone and nintedanib—are approved for the treatment of IPF, however they fail to reverse disease progression completely [6].

It is characterized by the chronic accumulation of extracellular matrix (ECM) in lung tissue, which gradually deforms the alveolar architecture, hinders gas exchange, and ultimately leads to death [3,7,8]. The pathophysiology is not clear; apparently, environmental exposures represent the main stimulus of damage in an aging dysfunctional lung epithelium. However, genetic and epigenetic factors are necessary to predispose the onset of the fibrotic process. Therefore, it has been proposed that the onset and development of IPF is multifactorial, since the synergy of various external and internal factors are necessary [7,9].

Fibroblasts are the key effector cells of IPF that, in response to profibrotic molecules present in the pulmonary microenvironment, are differentiated into myofibroblasts that synthesize and accumulate exacerbated components of the ECM in the pulmonary tissue, causing the clinical characteristics of the events [10]. Within the profibrotic molecules, RNA molecules have also been associated, both coding (mRNA) and different biotypes of noncoding RNA (ncRNA) [11,12]. Long ncRNAs (lncRNAs) are abundant and heterogeneous, with a length of more than 200 nucleotides [13]. lncRNAs have the ability to interact with other RNA biotypes, DNA, proteins, peptides, and some low molecular weight compounds, which allows them to participate in multiple molecular processes, including the regulation of gene expression at epigenetic, transcriptional, and post-transcriptional levels through different molecular mechanisms directly or indirectly [14].

For example, studies have suggested that the expression of lncRNA LINC01140 is upregulated in lung biopsies from patients diagnosed with IPF and in fibroblasts isolated from these patients. Furthermore, functional knockdown investigations have shown that LINC01140 acts as a positive regulator of proliferation in both control fibroblasts and those from IPF patients. Moreover, deletion of LINC01140 has been observed to result in an increased inflammatory response, particularly exacerbated in the context of IPF compared with control fibroblasts [15]. On the other hand, recent studies have shown an over-expression of lncRNA SNHG1 (lnc-SNHG1) in fibrotic lung tissues of murine models and in fibroblasts treated with TGF-β1. Furthermore, investigations involving manipulation of the functional expression of lnc-SNHG1 have revealed that its high expression facilitates fibroblast migration and invasion, as well as the secretion of molecules associated with fibrosis. In contrast, low expression of lnc-SNHG1 exerts opposite effects on these processes [16]. These findings imply that lncRNAs may play a significant role as regulators in the cellular and molecular processes underlying the development of IPF. Therefore, further research is required to delve deeper into these mechanisms and gain a better understanding of the pathogenesis of IPF.

Technological progress has led to the development of revolutionary tools such as RNA sequencing (RNA-seq) that have made it possible to explore the expression profiles of cells and tissues under physiological and pathological conditions. However, with increasing use, it has been observed that expression patterns are often inconsistent (even in studies of the same type) due to technical and biological variability, so it is necessary to implement studies that integrate data from multiple studies and allow a more detailed and integrated understanding of the cellular and molecular processes underlying this disease. In this study, we implemented a meta-analysis of three independent studies of RNA-seq data from pulmonary fibroblasts from IPF to identify profiles of lncRNA and mRNA by means of bioinformatics tools performing their functional characterization.

## 2. Results

### 2.1. Study Selection

**Figure 1:**
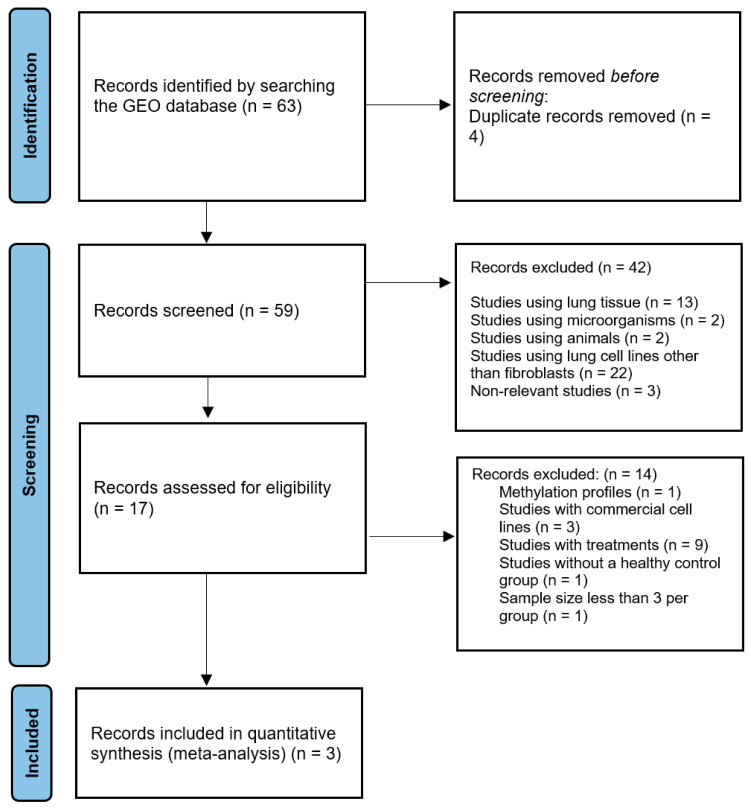
PRISMA flowchart for selecting eligible RNA-seq GEO datasets for transcriptional meta-analysis of primary fibroblasts isolated from lungs of IPF patients and their respective controls.

In the initial search, we collected 63 published high-throughput sequencing expression profiles published in the GEO database through 31 March 2022. We excluded 4 duplicates and 42 studies because they did not meet the inclusion criterion. The remaining 17 studies were further evaluated, and 14 studies were excluded, including 1 study based on methylation profiles, 3 studies using commercial cell lines, 9 studies using lung fibroblasts with treatments, 1 study lacking normal or healthy controls, and 1 study that had a sample size of less than 3 per group. Finally, three studies from the GEO database that met the inclusion criteria for the meta-analysis were selected (GSE99621, GSE180415, and GSE185492). The schematic flowchart of the meta-analysis is illustrated in Figure 1. 

In these 3 studies, study 1 (GSE99621) used the Illumina HiSeq 2500 platform (Homo sapiens) and contained 18 IPF samples and 8 controls; study 2 (GSE180415) used the Illumina HiSeq 4000 platform (Homo sapiens) and contained 5 samples of IPF and 4 controls; and study 3 (GSE185492) used the Illumina NovaSeq 6000 platform (Homo sapiens) and contained 12 IPF samples and 12 controls, obtaining a total of 35 IPF samples and 24 control samples (Table 1).

### 2.2. Differentially Expressed Gene

**Figure 2:**
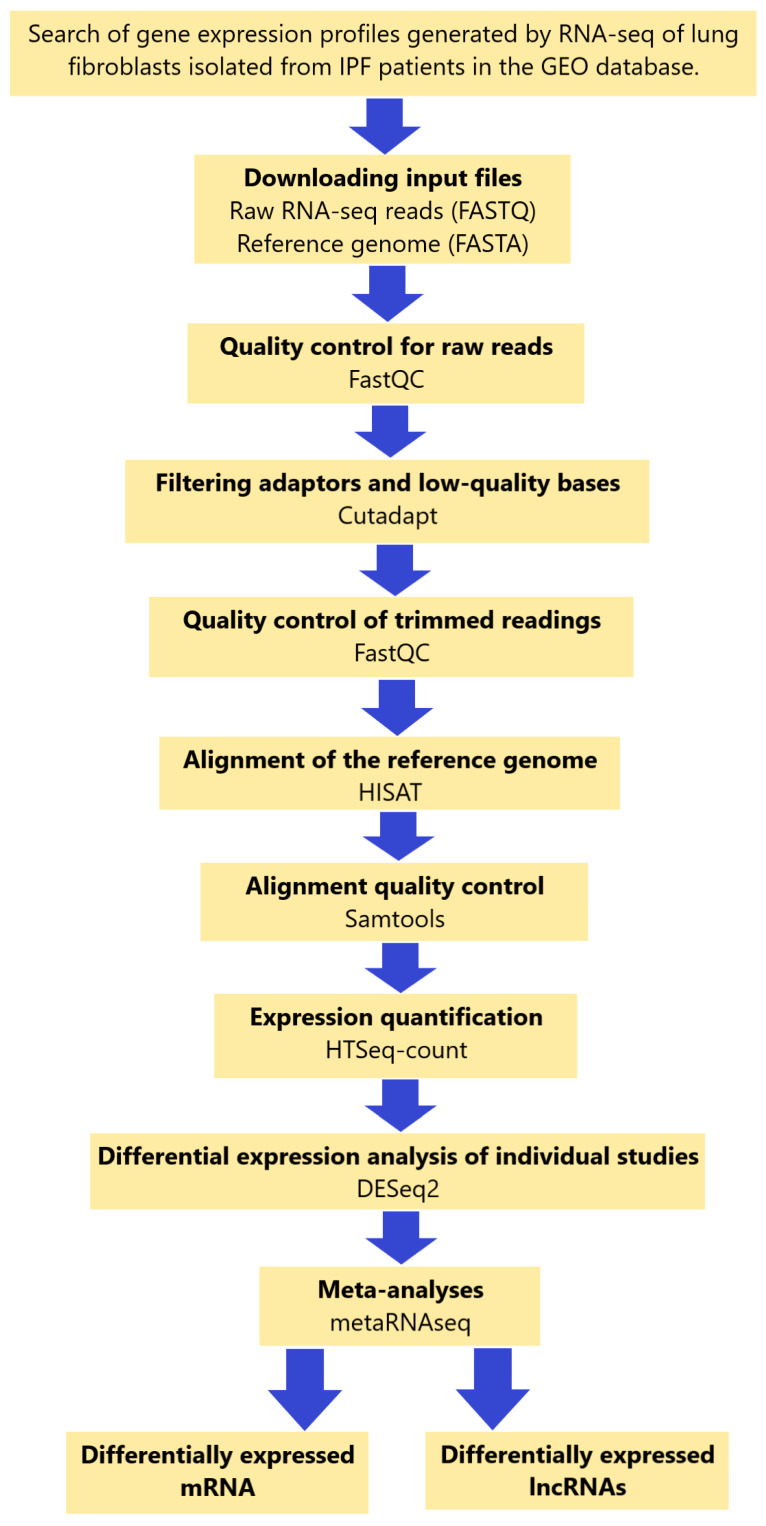
Illustrative diagram of the workflow for meta-analysis of GEO datasets from RNA-seq. The detailed processes are discussed in the Section 4.

**Figure 3:**
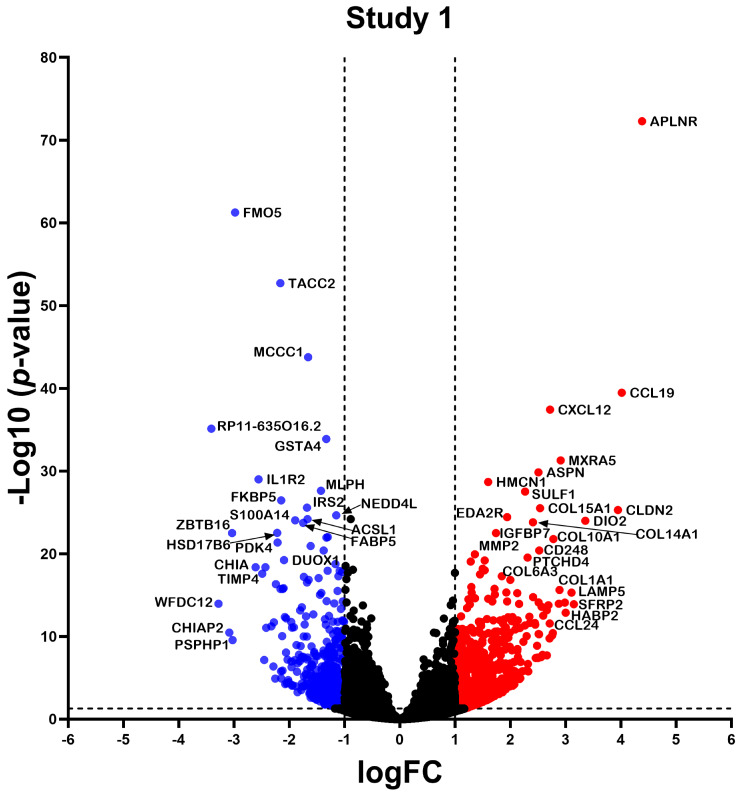
Differentially Expressed Genes in Study 1. The volcano plot illustrates the transcripts identified in study 1. Each dot on the graph represents a transcript; blue dots indicate underexpressed transcripts, red dots indicate overexpressed transcripts, and black dots represent transcripts with no significant differential expression. Differential expression was considered logFC > 1, and *p* < 0.05 was considered statistically significant.

**Figure 4:**
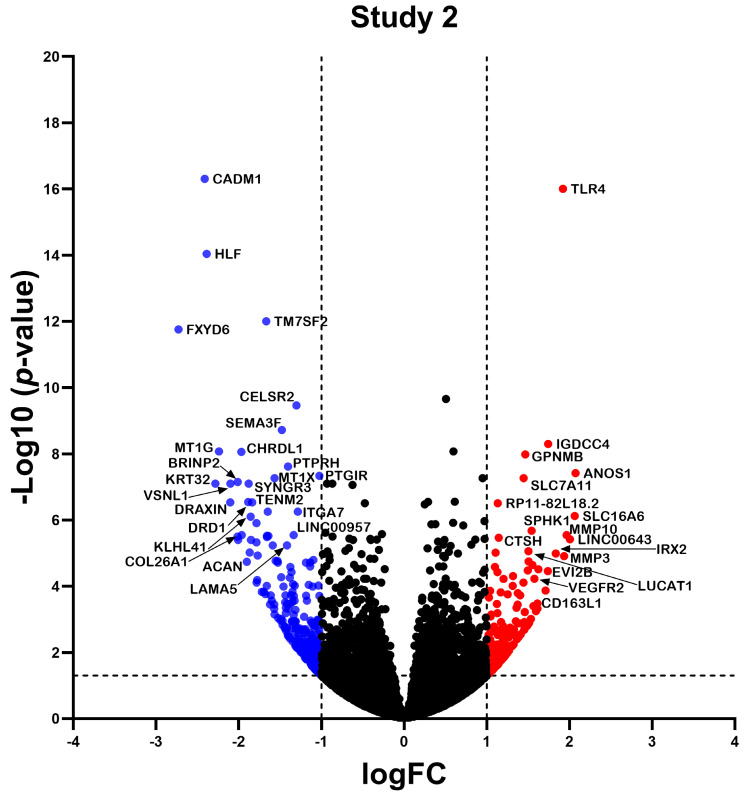
Differentially Expressed Genes in Study 2. The volcano plot illustrates the transcripts identified in study 2. Each dot on the graph represents a transcript; blue dots indicate underexpressed transcripts, red dots indicate overexpressed transcripts, and black dots represent transcripts with no significant differential expression. Differential expression was considered logFC > 1, and *p* < 0.05 was considered statistically significant.

**Figure 5:**
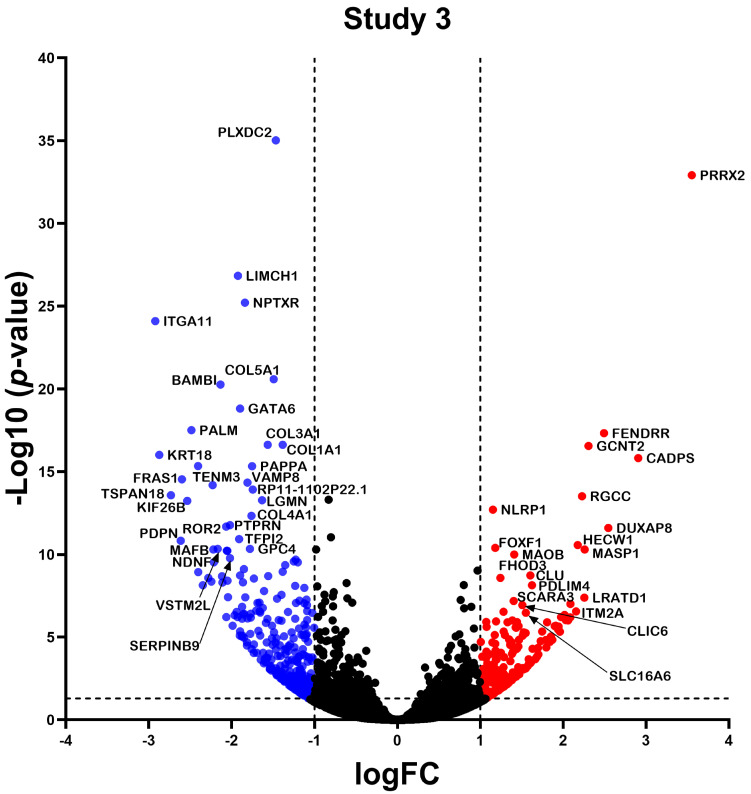
Differentially Expressed Genes in Study 3. The volcano plot illustrates the transcripts identified in study 3. Each dot on the graph represents a transcript; blue dots indicate underexpressed transcripts, red dots indicate overexpressed transcripts, and black dots represent transcripts with no significant differential expression. Differential expression was considered logFC > 1, and *p* < 0.05 was considered statistically significant.

RNA-seq data from the three studies were analyzed independently using a consistent bioinformatics workflow to better integrate the results and decrease the variability that the bioinformatics analyses might contribute (Figure 2). Study 1 identified a total of 1383 differentially expressed (DE) RNAs (DERNAs), of which 509 were under-expressed and 874 were over-expressed (Figure 3); study 2 identified a total of 440 DERNAs, of which 249 were under-expressed and 191 were over-expressed (Figure 4); and study 3 yielded 493 DERNAs, of which 290 were under-expressed and 203 were over-expressed (Figure 5).

### 2.3. Differential Expression Analysis of LncRNAs and mRNAs

**Figure 6:**
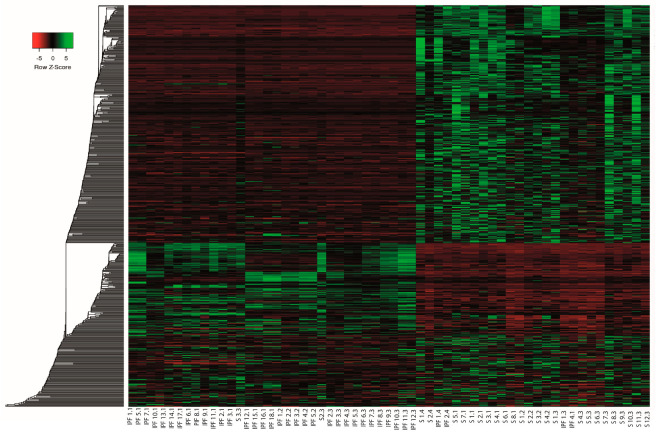
mRNA expression profiles. Heatmap of mRNA of z scores of hierarchical clustering expression of differentially expressed mRNAs in the IPF vs. CTL meta-analysis. Each row represents one mRNA and each column represents one sample. Red indicates low relative expression and green indicates high relative expression.

**Figure 7:**
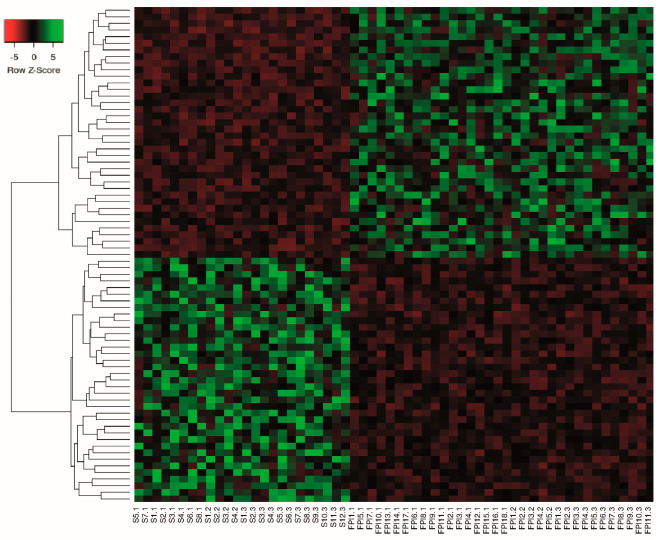
lncRNA expression profiles. Heatmap of lncRNA of z scores of hierarchical clustering expression of differentially expressed lncRNA in the IPF vs. CTL meta-analysis. Each row represents one lncRNA and each column represents one sample. Red indicates low relative expression and green indicates high relative expression.

Meta-analysis is a systematic method that allows the integration of several studies by increasing the number of samples, thereby increasing statistical power and reducing interstudy variability. Therefore, the DERNAs of each individual study were used to perform a meta-analysis using the MetaRNASeq package, which uses Fisher’s combined probability test. Through the meta-analysis, a total of 659 DERNAs were identified, of which 278 were under-expressed and 381 were over-expressed. We identified 252 under-expressed and 332 over-expressed mRNAs. For lncRNAs, we obtained 26 under-expressed and 49 over-expressed. To visualize the mRNA (Figure 6) and lncRNA (Figure 7) expression profiles obtained in the meta-analysis, heatmaps were generated, which showed that the expression profiles were different between samples from IPF and healthy individuals, indicating that, during IPF, there is a deregulation in the expression of mRNA and lncRNA. The complete list of differentially expressed mRNAs and lncRNAs can be found in Tables S1–S4.

### 2.4. PPI Network and Identification of Hub mRNAs

**Figure 8:**
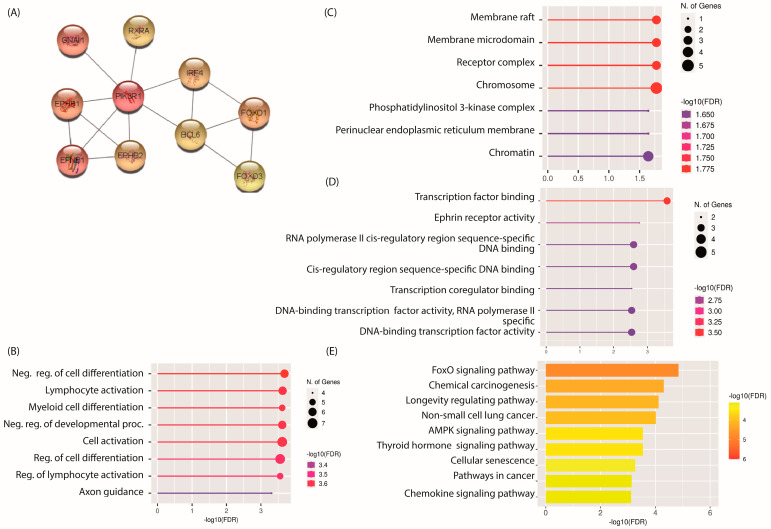
Identification and characterization of hub proteins. (**A**) Ten hub proteins identified by cytoHubba. Each node represents a protein, while each edge represents a protein–protein association. GO analysis of the ten hub proteins (**B**) PB—biological processes, (**C**) FM—molecular functions, and (**D**) CC—cellular components, (**E**) Kyoto Encyclopedia of Genes and Genomes (KEGG). The size of the circle represents the number of genes involved in each term, color represents significance, and FDR < 0.05 is considered statistically significant.

The gene products of mRNAs and proteins perform an enormous diversity of functions in cells through a set of direct (physical) and indirect (functional) interactions. Therefore, protein–protein interactions (PPIs) play central roles in cellular systems, such that the subtle alteration of PPIs can have major consequences and produce pathological phenotypes [17]. Moreover, PPI networks are extremely useful for predicting functions and identifying key molecules within large assemblies. The obtained PPI network contained all 584 proteins (nodes), of which 256 were part of the core network. However, 284 predicted functional associations (edges) were also identified (Figure S1). PPI networks are more powerful the greater the coverage of the interactome. However, the complexity of the network also increases, which makes its biological interpretation more difficult. Due to the above, the 10 central proteins within the PPI network were identified using Cytoscape and visualized by the complementary cytoHubba, which allowed us to identify with great precision the central proteins within a biological interactome network using 11 topological analysis methods [18]. The ten hub proteins identified by cytoHubba were PIK3R1, BCL6, GNAI1, RXRA, FOXO3, EPHB2, EFNB1, FOXO1, EPHB1, and IRF4, of which PIK3R1, BCL6, GNAI1, RXRA, EFNB1, FOXO3, and FOXO1 were identified as under-expressed in the meta-analysis, while EPHB1, EPHB2, and IRF4 were over-expressed (Figure 8A).

### 2.5. GO and KEGG Pathway Analysis of Hub mRNAs

To elucidate the biological significance of the ten identified hub proteins, GO analysis and KEGG pathway analysis were performed. GO analysis revealed that the ten hub proteins were involved in biological processes (BPs), such as negative regulation of cell differentiation, lymphocyte activation, myeloid cell differentiation, negative regulation of developmental processes, and cell activation (Figure 8B), while binding to transcription factors, Ephrin receptor activity, sequence-specific DNA binding of the cis-regulatory region of RNA polymerase ll, and binding to coregulators of transcription were identified among the major molecular functions (FMs) (Figure 8C). Cellular component (CC) terms revealed that these proteins were primarily localized in membrane lipid rafts, membrane microdomains, receptor complexes, and chromosomes (Figure 8D). In addition, analysis of KEGG pathways identified FoxO signaling pathways, chemical carcinogenesis, longevity regulatory pathways, and non-small cell lung cancer, as well as AMPK signaling pathways as major molecular functions (Figure 8E). The above results suggest that the ten hub proteins transcribed by mRNAs differentially expressed in lung fibroblasts from IPF localize to membrane raft mediating cell activation and differentiation via the FoxO and AMPK signaling pathways.

### 2.6. Hub Gene–Drug Interaction Network Analysis

**Figure 9:**
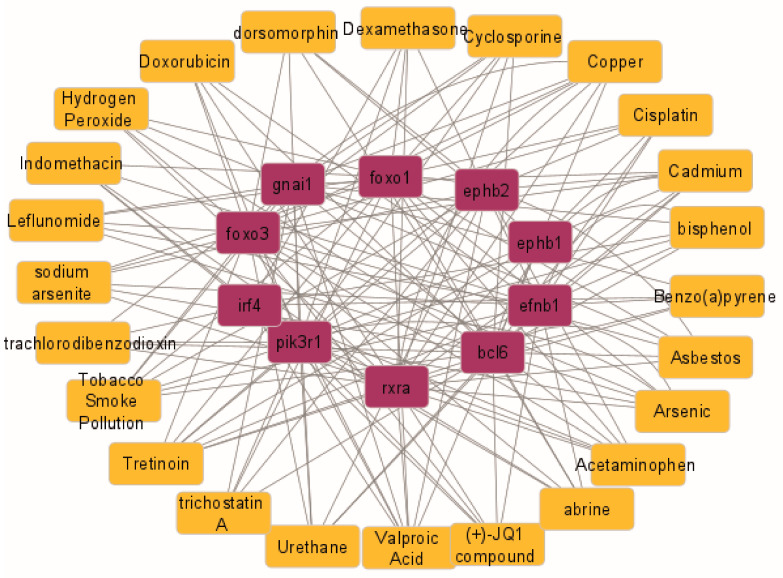
Toxicogenomics interaction network constructed with hub genes and drugs. The network is constructed from content curated by the comparative toxicogenomics database using Cytoscape. Purple rectangles correspond to hub genes, yellow rectangles correspond to toxicants, and lines represent toxicant–gene interactions.

Gene–environment interactions are postulated as triggers for different diseases in susceptible individuals. Due to the importance of these interactions, toxicogenomics has emerged, which allows the interpretation of the activity of genes and proteins in response to toxic substances [19,20]. To explore the interaction between hub genes and available drugs, a network was constructed using CTD and visualized by Cytoscape. The results identified abrine, acetaminophen, arsenic, asbestos, benzo(a)pyrene, bisphenol, cadmium, cisplatin, copper, cyclosporine, dexamethasone, dorsomorphin, doxorubicin, hydrogen peroxide, indomethacin, leflunomide, sodium arsenite, tetrachlorodibenzodioxin, tobacco smoke, tretinoin, trichostatin A, urethane, and valproic acid. (Figure 9).

### 2.7. Co-Expression of LncRNA/mRNAs

**Figure 10:**
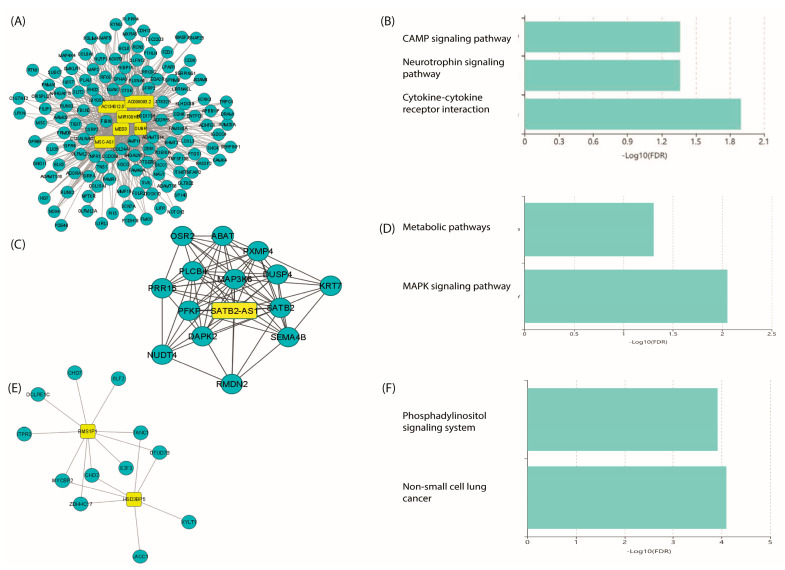
Identification and characterization of lncRNA. (**A**,**C**,**E**) Cluster identification is performed using the Cytoscape clusterMaker add-on. Yellow rectangles represent lncRNAs, blue circles represent mRNAs. (**B**,**D**,**F**) Most significant terms from KEGG pathways, FDR < 0.05.

lncRNAs have recently emerged as important regulators of IPF. However, functional characterization of lncRNAs is complicated because there are currently no databases with functional annotation of these transcripts; therefore, a commonly used strategy to infer the potential functions of lncRNAs is the construction of co-expression networks with mRNAs [21,22]. Based on the above, to better identify disease-related genes and lncRNAs, a weighted gene co-expression network analysis (WGCNA) was performed with all transcripts identified by meta-analysis based on Pearson’s correlation coefficient (PCC) using the WGCNA R package. WGCNA is a method used for finding highly synergistic expressed gene modules and the association between gene sets and disease [23]. The obtained co-expression network contained associations between 55 lncRNAs and 576 mRNAs (Figure S2). From the co-expression network, the top three clusters were identified, which were most densely connected to each other. Cluster 1 consisted of 6 lncRNAs (MSC-AS1, DUBR, MEG3, AC134312.5, AC009093.2, and MIR100HG) and 125 mRNAs (Figure 10A). Analysis of KEGG pathways revealed that the mRNA gene products included in this cluster were involved in cytokine–cytokine receptor interactions, cAMP, and neurotrophin signaling pathways (Figure 10B). Cluster 2 consisted of SATB2-AS1 lncRNA and 14 mRNAs (Figure 10C). KEGG analysis revealed that the mRNAs included in this cluster were involved in the MAPK signaling pathway and metabolic pathways (Figure 10D). Cluster 3 consisted of 2 lncRNAs (HSD3BP5 and BMS1P1) and 12 mRNAs (Figure 10E), which were found to be involved in the regulation of peptide hormone secretion, double-strand break repair, chromosome organization, and cellular response to damage stimulus (Figure 10F). Taken together, the results suggest that the lncRNAs identified in the meta-analysis are involved in a variety of biological processes, including cell adhesion, inflammatory response, compound metabolic processes, hormone regulation, and DNA double-strand break repair.

**Figure 11:**
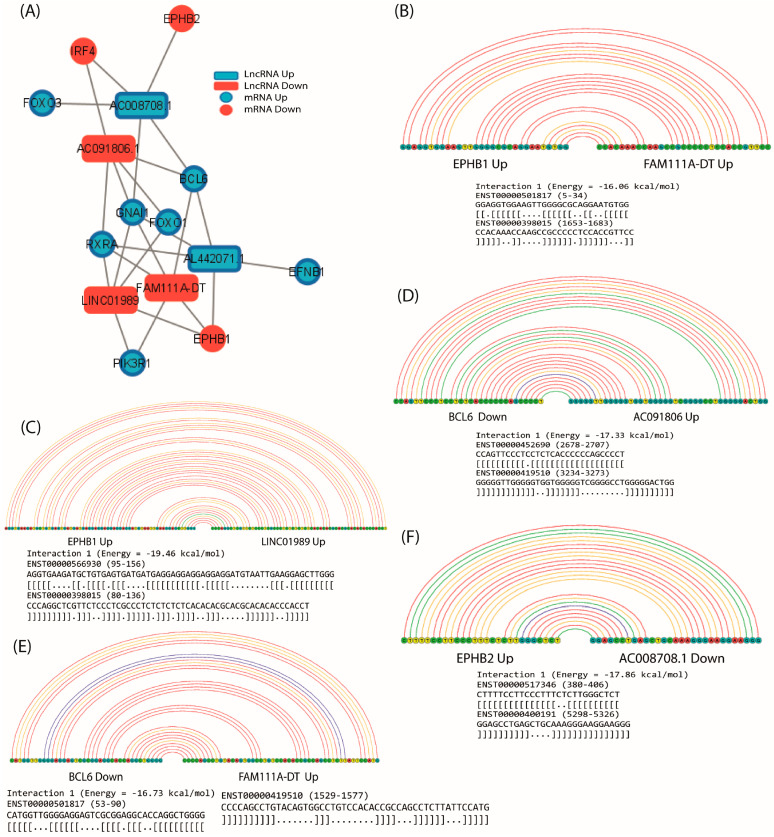
Co-expression regulation network and sequence interaction prediction analyses of hub mRNAs–lncRNAs. (**A**) Co-expression regulation network interaction analyses of hub mRNAs/lncRNAs. VARNA-based graphical representations obtained from LncRRIsearch (local base-pairing interactions as a function of the interaction energy between lncRNA and mRNA sequences). (**B**) EPHB1-FAM111A-DT interaction. (**C**) EPHB1-LINC01989 interaction. (**D**) BCL6-AC091806.1 interaction. (**E**) BCL6-FAM111A-DT interaction. (**F**) EPHB2-AC00870891 interaction. Circles represent nucleotides: yellow = thymine, green = cytokine, blue = guanine, and red = adenine. Lines represent base-pairing interaction.

Highly co-expressed groups are enriched in common biological functions and processes, and a high correlation may denote close regulatory proximity [24]. Because lncRNAs can regulate translation and transcription of mRNAs, a co-expression network was constructed with the ten hub mRNAs and all lncRNAs identified in the meta-analysis to identify lncRNAs that could be related to the ten hub mRNAs. The network identified 33 lncRNAs that were co-expressed with the 10 hub mRNAs (Figure S3). From the network, the five lncRNAs with the highest number of interactions (co-expression) were selected to identify a potential regulatory network between core mRNAs and lncRNAs. The results revealed that, of the five lncRNAs with the highest number of co-expressions, AC091806.1, FAM111A-DT, and LINC01989 showed under-expression in the meta-analysis, while lncRNAs AC008708.1 and AL442071.1 showed over-expression in the meta-analysis. These lncRNAs showed co-expression with the hub mRNAs (PIK3R1, BCL6, GNAI1, RXRA, EFNB1, FOXO3, and FOXO1), which were identified as under-expressed in the meta-analysis, while EPHB1, EPHB2, and IRF4 showed over-expression. The co-expression network revealed that AC008708.1 was co-expressed with BCL6, FOXO3, GNAI1, EPHB2, and IRF4. For its part, lncRNA AC091806.1 showed co-expression with IRF4, BCL6, FOXO1, GNAI1, and RXRA, and lncRNA AL442071.1 was co-expressed with BCL6, EFNB1, GNAI1, RXRA, and EPHB1. Furthermore, the lncRNA FAM111A-DT was co-expressed with EPHB1, BCL6, GNAI1, and PIK3R1, while the lncRNA LINC01989 was co-expressed with EPHB1, FOXO1, GNAI1, and PIK3R1 (Figure 11A). These findings suggest a possible involvement of lncRNAs AC008708.1, AC091806.1, AL442071.1, FAM111A-DT, and LINC01989 in the pathophysiology of IPF by regulating the expression of core mRNAs in IPF-associated fibroblasts.

### 2.8. Prediction of lncRNA–mRNA Interactions

One of the mechanisms by which lncRNAs can post-transcriptionally regulate mRNAs is by the direct binding of their sequences forming two-stranded lncRNA–mRNA structures that can promote or impede their translation or degradation [25]. Therefore, in order to elucidate whether co-expressed hub lncRNA–mRNA pairs could be regulated by interaction of their sequences, a comprehensive prediction analysis of the interaction between mRNAs and lncRNAs was performed using the LncRRIsearch web server, which performs RIblast prediction. RIblast predicts local base-pairing interactions as a function of interaction energy that is calculated using both accessibility energy and hybridization energy [26]. 

Interaction prediction analyses showed five energetically significant lncRNA–mRNA pairs: EPHB2-AC008708.1, BCL6-AC091806.1, BCL6-FAM111A-DT, EPHB1-FAM111A-DT, and EPHB1-LINC01989. The latter two were positively co-expressed while the others were negatively co-expressed (Figure 11B–F). These results suggest that AC008708.1, AC091806.1, FAM111A-DT, and LINC01989 lncRNAs can regulate EPHB2, EPHB1, and BCL6 mRNAs through direct interaction between their sequences.

## 3. Discussion

In the present study, we identified the hub mRNAs and lncRNAs present in pulmonary fibroblasts from IPF, as well as their functional characterization and adjacent mechanisms, through a meta-analysis of RNA-seq data and the implementation of in silico analysis. The advent of omics technologies has facilitated the understanding of diseases, since they allow for obtaining a large amount of biological information in a short period of time. The identification of differential expression profiles between two or more conditions has helped to understand the processes and functions that favor the development and progression of multiple diseases [27]. Our results allowed us to identify differential mRNA and lncRNA expression profiles of lung fibroblasts from IPF in 3 independent studies obtaining 2898, 962, and 1905 differentially expressed transcripts in each study, which demonstrates that, during IPF, there is a differential expression profile of mRNA and lncRNA in lung fibroblasts, which has been previously demonstrated by several studies in murine [28,29] and human fibroblasts [30,31].

With the implementation of NGS technologies, new problems have arisen related to the heterogeneity of results obtained due to the variability in techniques, instruments, and protocols, among others, which hinders the biological understanding of the results [32]. Therefore, a meta-analysis was performed based on the combination of the normal inverse and Fisher methods, with the integration of the 3 studies; a total of 659 differentially expressed transcripts were obtained, of which 584 were mRNAs and 75 were lncRNAs.

A limitation in our study arises from the assumption that transcript levels align with protein levels in the analyses of the PPI, KEGG, and toxicogenomics interaction networks. It is recognized that transcription levels frequently do not exhibit a strong correlation with the abundance of their respective protein products [33]. We speculate that, if the protein levels correspond to the mRNA levels, the assays could be met. The functional characterization of the differentially expressed mRNAs showed that their gene products were mainly located in plasma membrane components participating in cell communication, regulation of localization, and morphogenesis of anatomical structures through functions related to adhesion and binding to growth factors, cytokines, and hormones. These results could reflect the pathophysiology of IPF, in which fibroblasts physiologically participate in the morphogenesis and maintenance of pulmonary architecture through a series of ordered events. However, during IPF, a deregulation of biological processes influenced by growth factors, cytokines, and other biomolecules causes aberrant activation of fibroblasts that acquire greater migratory and biochemical capacity that contradictorily leads to the deterioration of pulmonary architecture [34].

We could also speculate that the protein–protein interaction network of the differentially expressed mRNA proteins allowed the identification of the following ten hub genes: BCL6, RXRA GNAI1, PIK3R1, FOXO1, FOXO3, EFNB1, EPHB1, EPHB2, and IRF4. GO analysis showed that their gene products are found in compartments such as chromatin and chromosomes, regulating the cellular response to cytokines, cell differentiation, DNA binding, and the activity of receptors and transcription factors. The enrichment of these terms may be attributed to the fact that the hub mRNAs code for proteins that are part of signaling pathways, most of which are well described in the context of IPF, particularly those related to the TGF-β pathway. It has been shown that the stimulation of fibroblasts with TGF-β1 decreases the expression level of BCL6, which is a transcriptional repressor that physiologically negatively regulates cell differentiation. Over-expression of BCL6 has been shown to reduce fibroblast proliferation and differentiation, because it is able to bind and negatively regulate SMAD4 [35]. BCL6 was found to be negatively regulated in the meta-analysis, which could indicate that its low expression could favor fibroblast differentiation. Nonetheless, our research has identified two noteworthy findings: (1) important signaling pathways are associated with fibrogenic processes in the lung and (2) toxicants are linked to fibrogenesis in the lung and in other organs or tissues. It is important to note that, while these results are significant, they do not necessarily provide conclusive evidence; rather, they underscore the importance of considering and validating genes and lncRNAs for future research. 

On the other hand, retinoid X receptor α (RXRA) has been found to negatively regulate the TGF-β promoter [36]. The direct relevance of RXRA in IPF is poorly understood, but it has been shown that, in liver fibroblasts, RXRA inhibits the expression of α-SMA and type I collagen [37]. In addition, it showed under-expression in the meta-analysis, which may suggest that its low expression does not inhibit TGF-β, allowing IPF fibroblasts to acquire an α-SMA+ phenotype and produce type I collagen.

One of the mechanisms involved in the transduction of extracellular stimuli is mediated by the interaction of receptors with one or more of the four major G-protein families. GNAI1 belongs to the Gαi family, whose members can regulate cell proliferation and differentiation [38]. GNAI1 was found to be under-expressed in IPF fibroblasts in the meta-analysis and has been observed in a murine model of pulmonary fibrosis [39]. It has also been shown that GNAI under-expression can favor tumor cell migration and invasion [35,40]. In IPF, GNAI has been considered a profibrotic regulator, although the mechanisms have not been elucidated [41].

The PI3K/AKT pathway has recently been considered a major regulator of IPF since it is able to regulate many disease-related functions, especially fibroblast to myofibroblast differentiation [42]. The PI3K/AKT pathway is involved in processes such as cell growth, proliferation, motility, metabolism, and survival [43]. Our meta-analysis showed that PIK3R1, a regulator of the PI3K pathway, was under-expressed, which can be explained by PIK3R1 encoding for the p85 protein, whose deletion leads to the activation of the downstream PI3K pathway. Therefore, its under-expression would favor PI3K signaling and thus the activation of fibroblasts [44,45]. 

The PI3K/Akt pathway is closely related to the FoxO family of transcriptional regulators, of which there are four isoforms: FOXO1, FOXO3, FOXO4, and FOXO6. These isoforms can act as transcriptional activators or repressors since they have a DNA binding domain that participates in a diversity of biological processes [46]. Our meta-analysis showed that FOX1 and FOX3 were under-expressed in IPF fibroblasts, which has been observed in fibrosis of different organs [47]. FOXO3 regulation appears to be related to the PI3K/Akt pathway, whereas FOXO1 is related to the WNT/β-catenin pathway [48,49].

In addition to the pathways classically related to IPF, ephrin ligand/Eph receptor signaling has been observed to act downstream of the TGF-β/SMAD3 pathway [50]. EFNB1 is a single-pass transmembrane protein that is part of the ephrin family, which includes cell surface ligands that can interact with Eph receptors (EPHB1, EPHB2). This interaction promotes activation of the Stat3 signaling pathway, which is intimately involved in fibrosis through fibroblast activation and differentiation [50,51]. Within the hub mRNAs, the ligand EFNB1 and the receptors EPHB1 and EPHB2 were identified. EFNB1 showed under-expression in the meta-analysis. However, there is little evidence to implicate the participation of EFNB1 in IPF. Nevertheless, due to the functions it performs, low expression could indicate a reduction in cell adhesion favoring cell migration [52]. EPHB1 and EPHB2 receptors were found to have over-expression in the meta-analysis. Studies of EPHB2 have shown that it is capable of promoting fibrosis through fibroblast activation and that it is over-expressed in the fibrosis of different tissues, although its participation in IPF is unknown [53,54].

The pathways enriched in the KEGG pathway analyses were consistent for over-expressed, under-expressed, and hub ten mRNAs, which were signaling, longevity regulation, and cancer-related pathways. The longevity regulatory pathways were relevant in the KEGG analysis, since IPF is an aging-related disease based on the accumulation of senescent cells. During IPF, fibroblasts show a senescent phenotype in which they are abnormally activated, and exhibit telomere shortening, metabolic reprogramming, mitochondrial dysfunction, and resistance to apoptosis; these features promote the onset and development of IPF [55]. Cancer pathways were another consistent term, which is not surprising, since IPF shares several common features and processes with lung cancer such as aberrant fibroblast proliferation, activation and differentiation, oxidative stress, and genetic and epigenetic variations [56,57,58]. 

Functional characterization of lncRNAs remains a challenge. Interestingly, our work allowed us to identify by lncRNA–mRNA co-expression networks the three most relevant clusters that could manifest the core functions of lncRNAs in IPF fibroblasts. Cluster 1 consisted of six lncRNAs over-expressed in IPF fibroblasts in the meta-analysis: lncRNAs MSC-AS1, DUBR, MEG3, AC134312.5, AC009093.2, and MIR100HG. Of the six lncRNAs, only MEG3 and MIR100HG have been evaluated in IPF, where they have shown high expression in epithelial cells [59,60]. 

Cluster 2 included SATB2-AS1 lncRNA, which was found to be over-expressed in IPF fibroblasts in the meta-analysis. Very little is known about its function; however, being an antisense transcript of SATB2 it is believed that it could play similar roles. Over-expression of the SATB2 gene has been shown to induce epithelial cell transformation and promote EMT, apparently through activation of the β-catenin pathway [61].

Cluster 3 included HSD3BP5 and BMS1P1, whose biological functions are unknown. However, KEGG pathway analysis of the three clusters demonstrated that these lncRNAs might be involved in cancer-related pathways, cytokine signaling, cAMP, MAPK, PI3K, and neurotrophin signaling pathways. It should be emphasized that the neurotrophin signaling pathway aims to transmit positive signals for cell survival and proliferation via the MAPK, PI3K, and phospholipase (PLC) pathways, and that this pathway has been implicated in multiple lung diseases, including pulmonary fibrosis [62]. Thus, the pathways identified are redundant with those identified with mRNAs, suggesting that the TGF-β, PI3K, FOXO, and MAPK pathways are highly relevant in IPF.

lncRNAs are involved in gene regulation at virtually all levels, including epigenetic regulation, nuclear and cytoplasmic trafficking, transcription, splicing, and mRNA translation [63]. Our results showed that 33 differentially expressed lncRNAs in IPF fibroblasts could regulate the ten hub mRNAs, as demonstrated by the co-expression network from which the five lncRNAs with the highest interaction were selected identifying AC008708.1, AC091806.1, AL442071.1, FAM111A-DT, and LINC01989, whose biological functions are poorly understood and currently none have been previously associated with fibrosis. However, our results suggest that they may regulate hub mRNAs at some level of gene expression, as they showed 25 co-expression pairs, 10 positively and 15 negatively correlated. Additionally, we identified that FAM11DT, LINC01989, AC008708.1, and AC091806.1 can physically interact by energetically favorable binding (<−16 Kcal/mol) of their sequences to EPHB1, EPHB2, and BCL6 and thus modulate their translation. The binding of lncRNAs to mRNAs can positively or negatively regulate their translation because they can bind to specific mRNA sequences regulating their stability and preventing or favoring degradation by exonucleases, miRNA binding, and binding to ribosome-binding proteins [64].

The toxicogenomic interaction network revealed a large number of toxicants that can negatively modulate the expression of hub mRNAs, some of which are widely related to pulmonary fibrosis, such as tobacco smoke, asbestos, hydrogen peroxide, arsenic, cadmium, and copper, which supports the importance of the hub mRNAs identified in this study [65,66]. In addition, drugs of clinical use such as dexamethasone, cyclosporine, acetaminophen, and leflunomide were identified, which is interesting since they are used with high frequency. In fact, dexamethasone is a corticosteroid that was initially used to treat patients with IPF. Currently, there is little evidence to support its use since it has been observed that its effect can be contradictory [67]. Acetaminophen and cyclosporine A have been considered etiologic agents of hepatic and renal fibrosis, respectively [68]. Although cyclosporine has also been used in combination with corticosteroids at low doses in patients with IPF, its effects have not been significant [69]. Similarly, leflunomide used for the treatment of rheumatoid arthritis has been contradictory in pulmonary fibrosis since it has shown both beneficial and detrimental effects [70,71]. The duality of the effects observed for these drugs in pulmonary fibrosis seems to indicate that their effect is dose dependent; however, more research is needed. The toxicogenomic network also included drugs used in chemotherapy such as doxorubicin and cisplatin. Chemotherapy carries the risk of acute exacerbations in IPF patients and contributes to an increased risk of mortality; therefore, the choice of chemotherapy should be made in consideration of risks and benefits [12].

## 4. Materials and Methods

### 4.1. Search and Selection Criteria for RNA-Seq Datasets

To investigate gene expression profiles of lung fibroblasts in patients with IPF, a search of the Gene Expression Omnibus database (GEO, https://www.ncbi.nlm.nih.gov/geo/, accessed on 1 April 2022) was conducted to identify relevant RNA-seq studies. The search was performed on publicly available gene expression datasets until 31 March 2022, using the keyword “idiopathic pulmonary fibrosis”. Search results were further narrowed through the following filters: entry type (“series”), study type (“expression profiling by high throughput sequencing”), and organism (“Homo sapiens”). The following data were extracted for each identified gene expression profile: GEO accession ID, sample size, platform, expression data, and references. The identified RNA-seq datasets were reviewed, filtered, and selected according to the following inclusion criteria:

Inclusion criteria:

1. Studies in primary fibroblasts isolated from human lung tissue.
2. Expression profiles generated by high-throughput sequencing (RNA-seq).
3. Comparative study between primary fibroblasts isolated from lung tissues of patients with IPF, as well as their respective controls; the latter consisting of primary fibroblasts isolated from normal human lung tissues.
4. Sample size equal to or more than 3 per group.
5. The raw RNA-seq-generated reads are publicly available.

Exclusion criteria:

1. Expression profiles generated by microarrays are not included.
2. Expression profiles from commercial cell lines are excluded.
3. Lung tissue samples are excluded.
4. Expression profiles from primary cultured fibroblasts isolated from species other than Homo sapiens are not considered.
5. RNA-seq profiles are not accepted if raw data are not publicly available.
6. Methylation profiles are excluded.

The meta-analysis was conducted following the guidelines provided in the 2020 Preferred Reporting Items for Systematic Reviews and Meta-Analyses (PRISMA) [72].

### 4.2. Data Collection

The acquisition of raw reads in FASTQ format was performed using Galaxy interface version 22.05 (https://usegalaxy.org, accessed on 5 April 2022). The reference genome was downloaded from GENCODE Version 32 (GRCh38.p13) in FASTA format.

### 4.3. Data Analysis of Individual Study Data

Data analysis was performed individually for each study on Galaxy platform version 22.05 (https://usegalaxy.org, accessed on 5 April 2022). The quality of raw reads was assessed with FASTQC, and bases with a Phred value ≤ 20 were considered poor quality and were removed with the Trimmomatic tool (version 0.38). Adapters were removed with the Cutadapt tool (version 1.16).

### 4.4. Alignment of Transcripts with the Reference Genome

Alignment was performed using HISAT2 (version 2.2.1). The human reference genome GRCh38 was used. The SAM format file was converted to its binary BAM format using samtools (version 1.12). Read counting of each transcript was performed using htseq (version 0.9.1).

### 4.5. Differentially Expressed Analysis 

Differential expression analysis between samples from healthy subjects and IPF was performed using DESeq2 (bioconductor-deseq2, version 1.22.1). Differentially expressed transcripts were screened as upregulated if they had a log2(FC) value ≥ 1 and an adjusted *p* value < 0.05, and downregulated if they had a log2(FC) value ≤ −1 and an adjusted *p* value < 0.05.

### 4.6. Meta-Analysis

Meta-analysis was performed using the R: metaRNASeq package, which is based on Fisher’s pooled probability using the following formula:

Transcripts with an adjusted *p* value > 0.05, transcripts with low expression levels, and transcripts with inconsistent expression directions were removed from the meta-analysis. The *p* values were adjusted for the Benjamini–Hochberg false discovery rate (FDR). An adjusted *p* value of less than 0.05 was considered statistically significant.

### 4.7. Gene Ontology and KEGG Pathway Enrichment Analysis

Gene ontology (GO), including biological processes (BPs), molecular functions (MFs), cellular components (CCs), and Kyoto Encyclopedia of Genes and Genomes (KEGG) analyses were performed using the R package ClusterProfiler version 3.5. The results were plotted with ggplot2 version 3.3.6, with terms and pathways with an FDR < 0.05 considered significant.

### 4.8. Protein–Protein Interaction Network

The protein–protein interaction network was performed on the Search Tool for the Retrieval of Interacting Genes (STRING database version 11.5 https://string-db.org/, accessed on 20 April 2022), considering a high confidence interaction score (0.700). Visualization of the interaction network was performed in Cytoscape software (version 3.9.1). MCODE was used to identify the four main clusters and cytoHubba was used to identify the ten hub genes.

### 4.9. Comparative Toxicogenomic Interaction Network 

A search for toxicant interactions was performed in the comparative toxicogenomics database (CTD) (http://ctdbase.org, accessed on 22 April 2022), selecting those chemicals that interacted with at least 5 mRNAs. The interaction network was visualized using Cytoscape.

### 4.10. lncRNA–mRNA Network Construction

Initially, the RNA expression data profiles of both genes and lncRNAs underwent rigorous testing to ensure the qualification of samples, genes, and lncRNAs. The co-expression analysis involved 75 lncRNAs and 584 mRNAs, where the Pearson correlation coefficient was calculated for each lncRNA-mRNA pair. This was accomplished using the normalized expression intensities and the WGCNA package within the RStudio interface (version 4.2.0), employing the “cor.test” function [23]. Subsequently, a weighted adjacency matrix was formulated using a power function represented by Amn = |Cmn| β (Cmn = Pearson’s correlation between gene/lncRNA m and gene/lncRNA n; Amn = adjacency between gene/lncRNA m and gene/lncRNA n). The lncRNA–mRNA pairs with correlation coefficients > 0.9 (positive) or <−0.9 (negative) were considered significant, the value of the soft-thresholding powers (β) was calculated in a range from 1 to 30, determining the value of β = 8. The topological overlap matrix (TOM), assessing the connectivity of a gene/lncRNA within the network, was defined as the sum of gene adjacency with all other gene/lncRNAs for the network gene/lncRNA ratio with a module size of 20. The co-expression network was visualized in Cytoscape software.

### 4.11. Prediction of lncRNA–mRNA Interactions

Base-pairing interaction prediction between lncRNA–mRNA sequences was performed using the WEB server LncRRIsearch (http://rtools.cbrc.jp, accessed on 25 April 2022), using as input the Ensembl identifier of each transcript, considering interactions with an energy threshold < −16 kcal/mol as significant.

## 5. Conclusions

In conclusion, the present study (based on bioinformatics analysis) identified differentially expressed hub lncRNAs (AC008708.1, AC091806.1, AL442071.1, FAM111A-DT, and LINC01989) and mRNAs (BCL6, EFNB1, EPHB2, FOXO1, FOXO3, GNAI1, IRF4, PIK3R1, and RXRA) from fibroblasts bearing IPF, and BCL6 may be directly regulated by AC091806.1 and FAM111A-DT, EPHB1 by FAM111A-DT and LINC01989, and EPHB2 by AC008708.1. Therefore, the present study may provide novel insights into the pathological mechanisms underlying IPF. However, further functional investigation is required to confirm these results.

## Figures and Tables

**Table 1:** Selected studies from the GEO database.

Study	ID_GEO	Platform	Sample Size	
			IPF	Control
Study_1	GSE99621	Illumina HiSeq 2500	18	8
Study_2	GSE180415	Illumina HiSeq 4000	5	4
Study_3	GSE185492	Illumina NovaSeq 6000	12	12
**Total sample size**			**35**	**24**

## Data Availability

Publicly available RNAseq datasets were analyzed in this study. These RNAseq datasets can be found in the GEO database (https://www.ncbi.nlm.nih.gov/geo/, accessed on 5 April 2024) with accession numbers GSE99621, GSE180415, and GSE185492.

## Supplementary Materials

The following supporting information can be downloaded at: https://www.mdpi.com/article/10.3390/ncrna10020026/s1, Figure S1: Protein–protein interaction network; Figure S2: Co-expression network mRNA-lncRNA; Figure S3: Co-expression network hub mRNA-lncRNA. Table S1: Complete list of overexpressed mRNAs; Table S2: Complete list of underexpressed mRNAs; Table S3: Complete list of over-expressed lncRNAs; and Table S4: Complete list of under-expressed lncRNAs.
