# Supplementary material for: lncRNA-mRNA Co-Expression and Regulation Analysis in Lung Fibroblasts from Idiopathic Pulmonary Fibrosis
Source: Noncoding RNA. 2024 Apr 17;10(2):26. doi: 10.3390/ncrna10020026 (PMC11054336; doi:10.3390/ncrna10020026)

Supplementary Figures

Figure S1. Protein-protein interaction network. The network was constructed using STRING with a high confidence minimum interaction score (0.700) and visualized using Cytoscape. Nodes forming part of the main interaction network are shown, disconnected nodes in the network were omitted, edges indicate functional and physical protein associations, line thickness indicates strength of data support

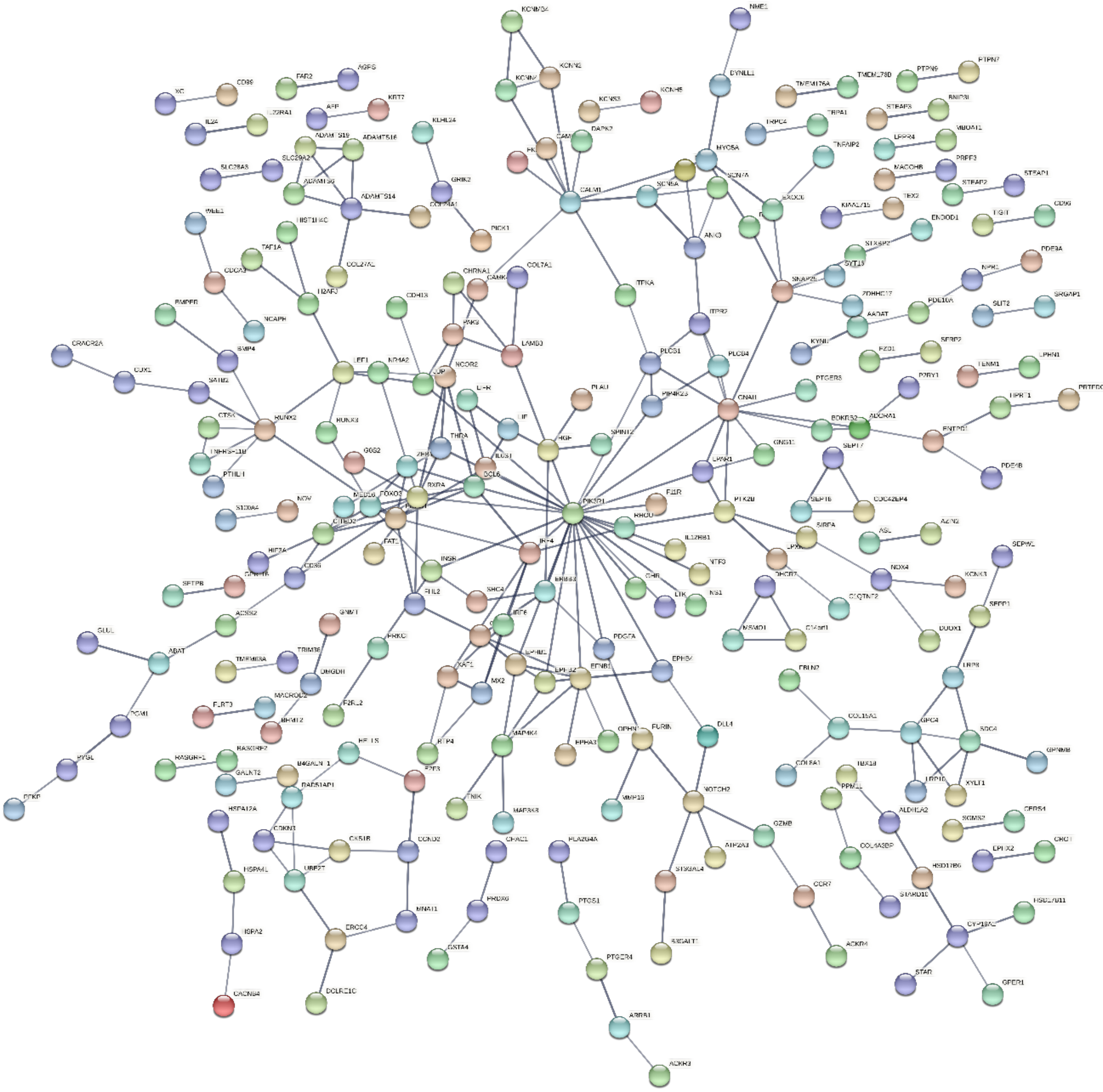

Figure S2. mRNA-lncRNA coexpression network. Yellow rectangles represent lncRNA, blue circles represent mRNA, correlation coefficients were calculated with the Pearson's quick correlation calculations function of the WGCNA package in R Studio, p> 0.9 (positive) or p< -0.9 (negative) were considered significant. The coexpression network was visualized using Cytoscape.

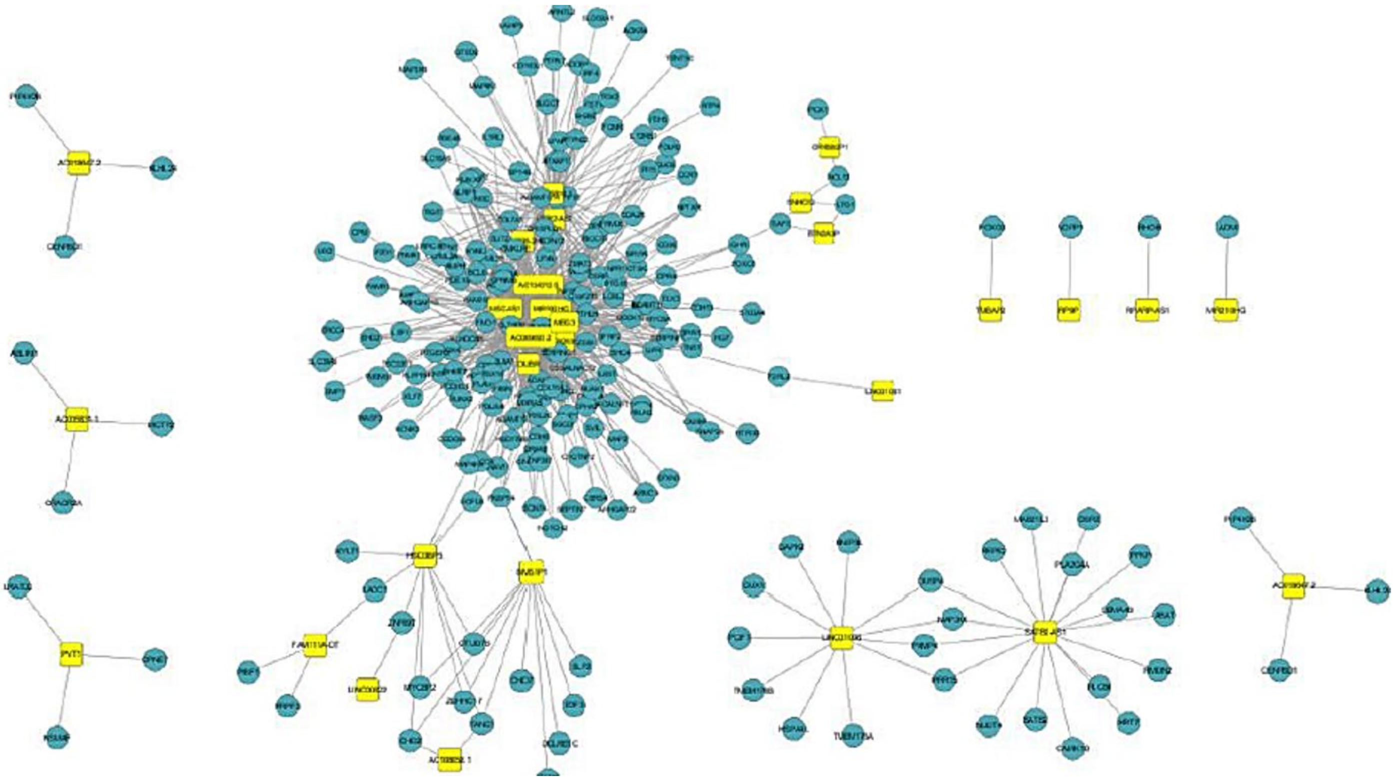

Figure S3. Figure S3. Coexpression network hub mRNA-lncRNA. Yellow circles nodes represent hub mRNAs, blue circles nodes represent lncRNAs, gray edges represent mRNA-lncRNA interaction, correlation coefficients were calculated with the Pearson's fast correlation calculations function of the WGCNA package in R Studio, p> 0.9 (positive) or p< -0.9 (negative) were considered significant. The coexpression network was visualized using Cytoscape.

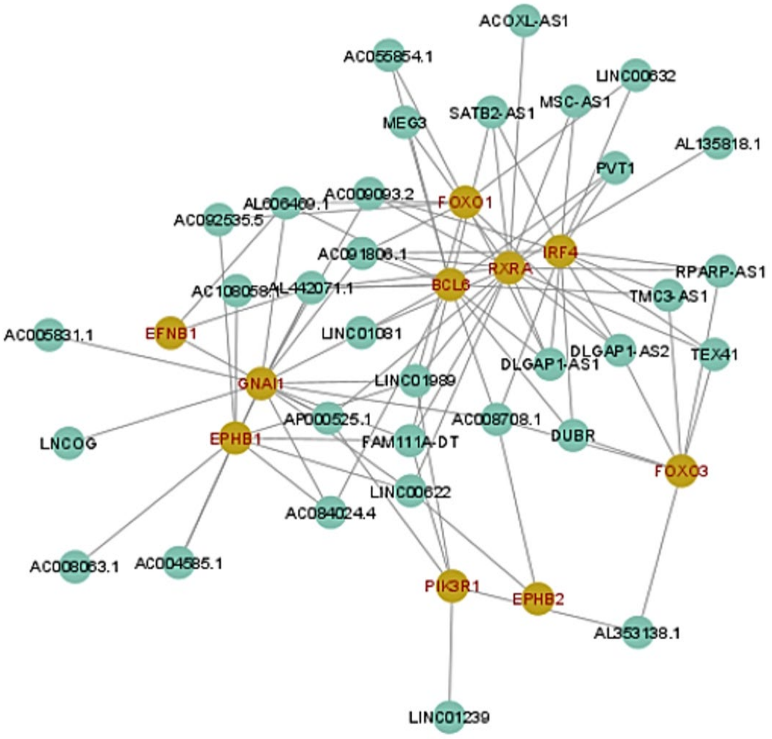

Supplement: Supplementary file 1 [file ncrna-10-00026-s001.zip › Supplementary Figures.pdf]
